# Aesthetics of everyday life and its related factors among older adults in Kashan (2021–2022)

**DOI:** 10.1186/s12877-023-04412-0

**Published:** 2023-11-22

**Authors:** Nafise Zamani, Fatemeh Sadat Izadi-Avanji, Azade Safa, Ismail Azizi-Fini, Esmaeil Mohammadnejad

**Affiliations:** 1 Department of Adults and Geriatric Nursing, School of Nursing, Kashan University of Medical Sciences, Kashan, IR Iran; 2 Department of Adults and Geriatric Nursing, Trauma Nursing Research Center, School of Nursing, Kashan University of Medical Sciences, Kashan, Iran; 3 Trauma Nursing Research Center, Kashan University of Medical Sciences, Kashan, Iran; 4 Department of Intensive care and Emergency Nursing, School of Nursing, Kashan University of Medical Sciences, Kashan, Iran; 5 Department of Medical-Surgical Nursing and Basic Sciences, School of Nursing and Midwifery, Tehran University of Medical Sciences, Tehran, Iran

**Keywords:** Aesthetics, Everyday life, Aging, Related factors

## Abstract

**Background:**

Aesthetics of everyday life are associated with the physical, mental, and social health of older adults, leading them to experience a successful old age. This study aimed to examine the aesthetics of everyday life and its related factors among older adults in Kashan from 2021 to 2022.

**Methods:**

This cross-sectional study consisted of 350 older adults who were referred to Urban Comprehensive Health Service Centers (UCHSC) in Kashan. Sampling was done by a two-stage method (cluster, random). The data collection was performed with a background information questionnaire and the Elderly’s Perception of Everyday Aesthetics scale (EPEA-S). Data were analyzed using an independent t-test, analysis of variance, Pearson’s correlation coefficient, and multiple linear regression tests in the SPSS software.

**Results:**

The mean age of the participants was 69.56 ± 6.63 years. The mean score of aesthetics of everyday life in older adults was 133.02 ± 14.73, with the family and others subscale receiving the highest score. The univariate test indicated a statistically significant correlation between age, employment status, education, income, smoking, social activities, physical activities, interest in artistic works, and the aesthetics of everyday life in older adults (P < 0.01). Multivariate linear analysis showed that age, employment status, smoking, income, social activities, physical activities, and interest in artistic works predicted and explained 28% of the variance of life aesthetics in older adults (R2 = 0.28).

**Conclusions:**

The aesthetics of everyday life of the Iranian older adults were in a good range. Healthcare providers and families of older adults can use this concept to enhance the elderly’s physical, mental, and social health.

## Background

Everyday aesthetics is a new branch of philosophical aesthetics that focuses on everyday events, setting and activities [1]. Before the 20th century, the scope of the subject of aesthetics was art-oriented and limited to various fields of fine arts [2]. But after that, three dominant trends were appeared in international aesthetics: aesthetics as a philosophy of art, natural aesthetics), and aesthetics of everyday life. These trends show that art, environment, and everyday life are the main topics of examining today’s aesthetics [3]. The important factor of focusing on everyday aesthetics is John Dewey’s view (1934) the American Pragmatist philosopher. Dewey in the book of “Art as experience”, has defined the aesthetics in the nature of experience, instead of putting aesthetic arena in objects or various situations [4]. In the aesthetics of daily life, the science of aesthetics extends to all dimensions of life and all experiences of human daily life [2]. Melchionne (2013) argue that everyday aesthetics is limited to aspects of human life that are characterized by widely shared routines or patterns. He considers five main areas for it: ‘food, wardrobe, dwelling, conviviality, and going out”. Almost everyone eats, wears clothes, lives somewhere, socializes and goes out to do something on a daily basis [5].

Martin Heidegger’s phenomenology, Ludwig Wittgenstein’s analytic philosophy, and John Dewey’s pragmatism are in some ways aligned with the aesthetics of everyday life. The philosophers’ discussions about aesthetics indicated their tendency to use aesthetics in everyday life [3]. For example, Wingstein mentions music as an expression of human life [6] or John Dewey believes that there is no clear boundary between art and everyday life. Experience, in its perfection in everyday life, becomes art [4].

Today, the aesthetics of everyday life have received a lot of attention, the reason of which is partly related to the recent developments in the world society [7]. The aestheticians believe that the aesthetics of everyday life are a reaction to the profound worldwide changes in contemporary culture and art that under influenced by it, aesthetic analysis has spread to all areas of living [3].

The everyday aesthetic reactions often guide people’s actions in the most direct way. Therefore, everyday aesthetics has a significant power to influence the quality of life [1]. In old age, the aesthetics of everyday life shapes the way older adults behave and deal with the challenges they face. The adoption of the aesthetics of life by the elderly helps them to be linked with society, actively participate in their favorite activities [8]. Furthermore, in the physical dimension, it can improve the older adults’ motivation to self-care and management of chronic diseases [9]. Many studies have examined the relationships between aesthetics and happiness or one’s life satisfaction. Their results showed that the aesthetic of everyday life increase happiness and life satisfaction in the older adults [10–13].

Older adults’ population in Iran is growing rapidly. One of the concerns of the health system is to enhance the quality of life and well-being of the older adults [14]. Welfare and well -being is partly dependent on the aesthetic of everyday life. In fact, there may be features of everyday aesthetic life (e.g., autonomy, flexibility) that make it useful for well-being [5]. Therefore, it is necessary to increase the knowledge of health care providers on the older adults’ aesthetic of everyday life in different cultures [15]. This study was aimed at examining the aesthetics of everyday life and its related factors among older adults in Kashan from 2021 to 2022. This study can provide a valuable insight into the aesthetic of everyday life of the older adults to maintain their health and well -being.

## Methods

### Design

This cross-sectional descriptive study aimed to examine the aesthetics of everyday life and its related factors among older adults in Kashan from 2021 to 2022.

### Participants

The study population was people aged 60 years and older. Inclusion criteria were Iranian people aged 60 years and older who wanted to participate in the study, were able to communicate verbally, and had no mental illness and cognitive disorders (a score higher than 20 from the MMSE), while the exclusion criterion was an incomplete questionnaire.

The literature review suggested no study similar to the present study, so a pilot study was conducted using the following formula to calculate a quantitative variable in a population. The sample size was 320 (δ = 14.6, d = 1.6), but 350 individuals were considered to increase the study rigor.

Sampling was done in two stages: first, 10 centers were selected randomly among 21 Urban Comprehensive Health Service Centers in Kashan (UCHSC). The sample size of each center was determined according to the total number of older adults covered by the same center. Then, the health records of the older adults in each center were numbered and the desired numbers were randomly selected. The first researcher contacted the eligible samples to explain the study objectives and invited them to the center to complete the questionnaires. She interviewed with the older adults who were unable to read and write.

### Data collection

The data were collected using a background information questionnaire and the Elderly’s Perception of Everyday Aesthetics scale (EPEA-S). The background information questionnaire included age, sex, marital status, employment status, education level, income level, number of children, living arrangement, underlying disease, physical activities, social activities, interest in artistic works, and walking in nature.

The EPEA-S was developed by Izadi et al. in 2021. The questionnaire consisted of 34 items and 7 subscales, including family and others (8 items), art and artistic activities (5 items), communication and social presence (5 items), spirituality and transcendence of the soul (5 items), beauty of appearance and physical health (4 items), independence in life (4 items), and perception of the environment beauty (3 items). The items were rated on a 5-point Likert scale (from strongly disagree = 1 to strongly agree = 5) and none of them received reverse scores. The scores ranged from 34 to 170, with a higher score indicating a greater perception of the life aesthetics. The scoring level of the questionnaire was divided into four 25% quartiles (i.e. bad, average, good, and excellent). The internal consistency for the whole scale and subscales was 0.92 and 0.90 − 0.66 using Cronbach’s alpha coefficient. The intraclass correlation coefficient (ICC) of 0.9 was estimated between the test and retest scores [16].

### Data analysis

The data normality was confirmed using the Kolmogorov-Smirnov test. Frequency and mean were used for descriptive statistics. In univariate analysis, an independent t-test, one-way analysis of variance (ANOVA), and Pearson’s correlation coefficient were used to determine the relationship between perception of the aesthetics of everyday life and its related factors. A multiple linear regression test was used to determine the factors predicting of the aesthetics of everyday life in older adults. The significance level was considered 0.05. The data were analysis using the SPSS software, version 16.

### Ethical consideration

The study was approved by the ethics committee of Kashan University of Medical Sciences with the code of ethics No: IR.KAUMS.NUHEPM.REC.1399.068. All ethical codes and principles related to the study were followed according to the Declaration of Helsinki. The researcher explained the study objectives to the participants, received their written informed consent and assured them that their information would remain confidential and the research results would be made available to them if they wished. For illiterate participants, informed consent to participate was obtained from their legal guardians. The researcher observed all health protocols during the COVID-19 pandemic when collecting data.

## Results

The mean age of the older adults was 69.56 ± 6.63 years (60–91 years). Most of them were male (54.9%), married (77.1%), had degrees below diploma (65.7%) and had no basic insurance (37.4%). The mean number of children was 4.58 ± 2.12. Table 1 summarized other background information.

**Table 1:** Characteristics of the older adults participating in the study

Variables	Level	Number	Percentage
Employment status	Employed	31	8.9
	Retired	146	41.7
	Housekeeper	148	42.3
	Disabled	25	7.1
Education level	Illiterate	41	11.7
	Middle/high school	230	65.7
	Diploma/higher	78	22.6
Income level	Insufficient	107	30.67
	Sufficient	243	69.4
Living arrangement	Alone	64	18.3
	Living with spouse	228	65.1
	Living with children	58	16.6
Smoking	Yes	40	11.4
	No	310	88.6
Walking in nature	Yes	264	75.4
	No	86	24.6
Social activities	Yes	185	52.9
	No	165	47.1
Membership in groups	Yes	246	70.3
	No	104	29.7
Physical activities	Yes	69	19.7
	No	281	80.3
Interest in artistic works	Yes	148	42.3
	No	202	57.7

The ANOVA test indicated a significant difference in the mean scores of aesthetics of everyday life and employment status (p = 0.001, F = 5.86), while Tukey’s post hoc test showed a significant difference in the mean scores of aesthetics of everyday life and other occupational groups. The ANOVA test showed a significant difference in the mean scores of aesthetics of everyday life and education level (p = 0.001, F = 7.70), while Tukey’s post hoc test indicated a significant difference in the mean scores of aesthetics of everyday life between older adults with a diploma and those with other degrees. The statistical t-test showed a significant difference in the mean scores of aesthetics of everyday life, income level (P = 0.01, t=-2.45), smoking (P = 0.004, t=-2.91), social activities (P = 0.001, t = 3.38), physical activities (P = 0.0001, t = 4.20), and interest in artistic works (P = 0.0001, t = 8.11) (Table 2).

The t-test showed no significant difference in the mean scores of aesthetics of everyday life, gender (P = 0.09), marital status (P = 0.40), underlying disease (P = 0.31), walking in nature (P = 0.68). ), and membership in groups (P = 0.41). The ANOVA test did not show a significant difference in the mean scores of aesthetics of everyday life and the living arrangement (p = 0.29, F = 1.22).

**Table 2:** Relationship between the aesthetics of everyday life with characteristics of the older adults

Variables	level	Mean ± SD*	P-value
Employment status	Employed	141.83 ± 16.02	P = 0.01, F = 5.86^††^
	Retired	130.34 ± 15.54	
	Housekeeper	134.06 ± 12.88	
	Disabled	131.56 ± 14.35	
Education level	Illiterate	128.58 ± 13.84	P = 0.001, F = 7.70^††^
	Middle/high school	132.00 ± 13.99	
	Diploma/higher	± 16.01 138.29	
Income level	Insufficient	130.13 ± 13.41	P = 0.01, t= -2.45^†^
	Sufficient	134.29 ± 15.12	
Smoking	Yes	126.70 ± 14.68	P = 0.004, t= -2.91^†^
	No	133.83 ± 14.56	
Social activities	Yes	135.50 ± 14.67	P = 0.001, t = 3.38^†^
	No	130.24 ± 14.33	
Physical activities	Yes	139.55 ± 15.50	P = 0.0001, t = 4.20^†^
	No	131.41 ± 14.11	
Interest in artistic works	Yes	139.87 ± 12.64	P = 0.0001, t = 8.11^†^
	No	128.00 ± 14.14	

^††^ANOVA test, ^†^ Independent t‑test, *SD: standard deviation

The Pearson’s correlation coefficient showed a negative and significant correlation between age and aesthetics of everyday life among older adults (p = 0.001, r=-0.18).

The study results showed that the mean score of aesthetics of everyday life among older adults was 133.02 ± 14.73, with 11.1% (39) of the participants receiving moderate scores, 68.3% (239) of the participants receiving high scores, and 20.6% (72) received a very high score for perception of the life aesthetics among older adults. The older adults in this study received no low and very low scores for aesthetics of everyday life among older adults. Table 3 shows the mean (standard deviation) scores for aesthetics of everyday life and its subscales among older adults, with family and surrounding people, and spirituality and self-exaltation receiving the highest scores, respectively.

**Table 3:** The mean score of aesthetics of everyday life and its subscales in the older adults

Subscales of perception of the life aesthetics among older adults	Minimum	Maximum	Mean ± SD*	Mean score/100
Family and others	8	40	35.3 ± 49.70	85.91
Art and artistic activities	5	25	14.5 ± 92.15	49.62
Communication and social presence	5	25	19.3 ± 28.41	71.40
Spirituality and transcendence of the soul	5	25	21.2 ± 86.79	84.30
Beauty of appearance and physical health	4	20	16.2 ± 63.24	60.80
Independence in life	4	20	13.3 ± 72.48	78.96
perception of the environment beauty	3	15	11.2 ± 10.67	67.50
Total score of perception of the life aesthetics among older adults	34	17	133.14 ± 02.73	72.81

*SD: standard deviation

The stepwise regression showed that age, employment status, smoking, income, social activities, physical activities and interest in artistic works predicted and explained 28% of the variance of perception of the life aesthetics among older adults (R2 = 0.28) (Table 4).

**Table 4:** Stepwise regression analysis of characteristics of older adults with aesthetics of everyday life

Variables	P-value	T	Beta	Adjusted R2	R2
Age	0.013	-2.485	− 0.255	0.28	0.29
Smoking	0.006	2.761	5.933		
Social activities	0.006	-2.778	-3.859		
Income	0.007	2.723	4.082		
Employment status	0.0001	3.810	9.064		
Physical activities	0.007	-3.025	-5.259		
Interest in artistic works	0.0001	-8.369	-11.431		

## Discussion

This study aimed to explain the aesthetics of everyday life and its related factors among older adults. The aesthetics of everyday life of the Iranian older adults were in a good range. Aesthetics is associated with many life experiences, including interactions with other people and a range of everyday activities such as eating, exercise, etc. [1]. The review of the literature suggested no study that directly investigated this issue in old age. Therefore, the study results are indirectly related to aesthetics. For example, the studies conducted on happiness addressed the components of aesthetics, art and, sports.

Zarepour Moghadami in Tabriz (2022) reported a high level of aesthetics among older adults [17]. The results of a qualitative study also showed a correlation between aesthetics and happiness among older adults, and inner beauty (character, love, respect for others, belief in God and caring for the soul, mutual behavior and, ethics) was more important than outer beauty (appearance). Persistent happiness is the result of balancing inner and outer beauty [11]. A study in Singapore reported moderate to high happiness among 96% of older adults [18].

The study results indicated that the subscale of family and others received the highest score. This subscale consisted of two categories: loving family and surrounding people and receiving goodness from them. The aesthetics of everyday life have no definite boundaries. For example, aesthetics in the living environment can have two aspects, the natural aspect and the social aspect, including belonging and emotions that are exchanged in daily life [4]. Previous studies supported this result and found that the communication of older adults with their families, children, neighbors and other people was an important factor in improving their physical, mental and social health, especially in dealing with life stresses [19, 20]. Shapero et al. (2018) also demonstrated that showing kindness and love to older adults improved their cognitive functions and flexibility [21]. The studies suggest that interaction with surrounding people and a strong social network stimulate brain activity, reduce the risk of dementia, have protective effects, improve health and quality of life and prevent cognitive decline among older adults [22, 23]. Therefore, family as a vital source of support helps older adults to maintain and continue their social interactions and protects them from loneliness, social isolation and depression.

Spirituality and transcendence of the soul was the second subscale with the highest score. Spiritually caused older adults to achieve peace of mind and better adapt to life problems [24, 25], but the results of a study showed no significant relationship between spirituality, resilience and adaptation [26]. This inconsistency can be due to the difference in age and culture of the participants. Although, God is the source of all beauties in different religions and cultures, worshiping and connecting to a higher power leads to greater communication with God and a better perception of beauty.

The study results showed a decreased level of aesthetics in old age. Older adults face sensory disorders, especially in sight and hearing, which progress slowly and gradually [27], as well as muscular and skeletal problems that can limit their mobility. Therefore, they are unable to walk in nature and use the beauty of the surrounding environment [28], which reduces their perception of the surrounding aesthetics. Seeing a beautiful view is not just a visual thing. It also has a positive effect on the human soul and body by creating peace and a sense of dreaming and helps to improve the level of health [2]. The study results showed that although married people received higher scores of aesthetics of everyday life than single, widowed older adults, they were not statistically significant. One study compared the happiness of older women with and without a spouse and showed that marriage had no significant relationship with happiness in these people [29], but another study (2022) found that married older adults were happier [17]. Celik et al. in Turkey (2018) revealed that married older people were more satisfied with life aesthetics [30]. Therefore, we require more studies in this area.

In the present study, older adults living with people other than their spouses and children reported lower scores for aesthetics of everyday life. The results of studies show that the place of residence of older adults had a major impact on their happiness [31, 32]. For example, older adults living in nursing homes reported lower levels of happiness than those living with their family members [31]. Despite the increase in older adults living alone, living with spouses and children is still the dominant pattern in Iran [33]. This issue can be one of the reasons that aesthetics is involved with the basic rhythms of life in the East [4]. In general, living with family members and receiving their social support both can have a protective effect on the stress of old age [20] and lead to a better aesthetics of everyday life among older adults.

The study results indicated that employed older adults reported a higher aesthetics of everyday life than other older people (housekeepers, retired or disabled ones) because they could participate in social activities. In addition, the older adults with a high income reported a higher aesthetics of everyday life. One study found that people who were satisfied with their incomes were happier than those who were dissatisfied [34]. It seems that a good income can make it easier to use resources and enjoy life more, so older adults with a high income have a greater aesthetic of everyday life.

The study results showed a significant difference in scores of aesthetics of everyday life among older adults who participated in social and physical activities and were interested in artistic works, so they were more satisfied with life [22]. A study indicated that older adults’ active participation in social activities, leisure time and physical activities made them more satisfied with life and prevented functional problems related to age [35]. Another study found that recreational activities had a significant effect on improving people’s perception of their health, independence, lifestyle, life expectancy and quality of life [36]. Yet another study indicated that physical activity and fun increased older adults’ self-confidence and satisfaction and protected them from feeling unimportant [37]. These results suggest the importance of recreational activities in maintaining older adults’ health. The results of one study showed that older adults felt valuable when communicating with others and had more motivation to live [38]. Therefore, the older adults who are more involved in social, physical and artistic activities have a greater aesthetics of everyday life.

The aesthetics of everyday life can have positive effects on the physical, mental and social health of older adults. Human takes refuge in beauty to get rid of the problems and hardships of everyday life. Aesthetics has a significant power that leads to correct actions of people in the direction of positive interaction and adaptation to life and can cause positive effects on the quality of a person’s life [4, 39].

## Conclusion

The aesthetics of everyday life of the Iranian older adults was in a good range. Nurses and families of older adults can use this concept and its predictor variables in nursing care plans to enhance the elderly’s physical, mental, and social health and have a successful old age. Involving older adults in artistic activities and physical activities, designing beautiful and pleasant environments for the lives of older adults, and increasing their social participation can be among these actions. As this is the first cross-sectional study that addressed the aesthetics of everyday life among older adults, it is suggested to conduct more studies in other settings. In addition, it is recommended to design interventions in this field to increase the aesthetics of everyday life in older adults.

## Data Availability

The datasets generated during or analyzed during the current study available from the corresponding author on reasonable request.

## Abbreviations

UCHSC: Urban Comprehensive Health Service Centers
EPEA-S: Elderly’s Perception of Everyday Aesthetics scale
ICC: Intraclass Correlation Coefficient
